# Construction and Evaluation of Rodent-Specific rTMS Coils

**DOI:** 10.3389/fncir.2016.00047

**Published:** 2016-06-30

**Authors:** Alexander D. Tang, Andrea S. Lowe, Andrew R. Garrett, Robert Woodward, William Bennett, Alison J. Canty, Michael I. Garry, Mark R. Hinder, Jeffery J. Summers, Roman Gersner, Alexander Rotenberg, Gary Thickbroom, Joseph Walton, Jennifer Rodger

**Affiliations:** ^1^Experimental and Regenerative Neurosciences, School of Animal Biology, University of Western AustraliaPerth, WA, Australia; ^2^Departments of Communication Sciences & Disorders and Chemical & Biomedical Engineering, University of South FloridaTampa, FL, USA; ^3^Global Center for Hearing and Speech Research, University of South FloridaTampa, FL, USA; ^4^School of Physics, University of Western AustraliaPerth, WA, Australia; ^5^Wicking Dementia Research and Education Centre, University of TasmaniaHobart, TAS, Australia; ^6^Human Motor Control Lab, School of Medicine, University of TasmaniaHobart, TAS, Australia.; ^7^Research Institute for Sport and Exercise SciencesLiverpool John Moores University, UK; ^8^Department of Neurology, Boston Children’s Hospital, Harvard Medical SchoolBoston, MA, USA; ^9^Burke-Cornell Medical Research InstituteWhite Plains, NY, USA

**Keywords:** rTMS, rodent models, magnetic field, electric field, motor evoked potentials

## Abstract

Rodent models of transcranial magnetic stimulation (TMS) play a crucial role in aiding the understanding of the cellular and molecular mechanisms underlying TMS induced plasticity. Rodent-specific TMS have previously been used to deliver focal stimulation at the cost of stimulus intensity (12 mT). Here we describe two novel TMS coils designed to deliver repetitive TMS (rTMS) at greater stimulation intensities whilst maintaining spatial resolution. Two circular coils (8 mm outer diameter) were constructed with either an air or pure iron-core. Peak magnetic field strength for the air and iron-cores were 90 and 120 mT, respectively, with the iron-core coil exhibiting less focality. Coil temperature and magnetic field stability for the two coils undergoing rTMS, were similar at 1 Hz but varied at 10 Hz. Finite element modeling of 10 Hz rTMS with the iron-core in a simplified rat brain model suggests a peak electric field of 85 and 12.7 V/m, within the skull and the brain, respectively. Delivering 10 Hz rTMS to the motor cortex of anaesthetized rats with the iron-core coil significantly increased motor evoked potential amplitudes immediately after stimulation (*n* = 4). Our results suggest these novel coils generate modest magnetic and electric fields, capable of altering cortical excitability and provide an alternative method to investigate the mechanisms underlying rTMS-induced plasticity in an experimental setting.

## Introduction

**FIGURE 1:**
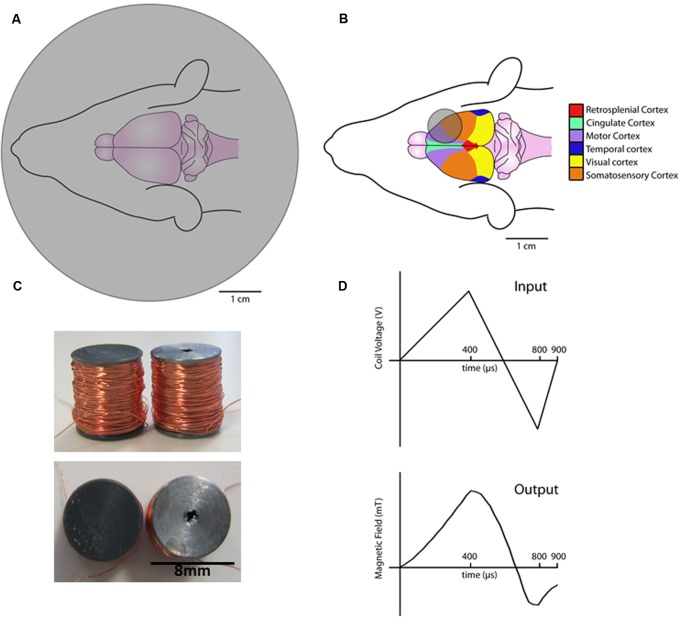
**Schematic diagrams of coils and waveforms.** Commercial 70 mm round coil over a rat brain **(A)**. Rodent-specific 8 mm round coil placed over the rat brain **(B)**. Birdseye (top) and side on views (bottom) of the novel air-core coil (left) and iron-core coils (right) **(C)**. Diagram of the input coil voltage (top) and resulting magnetic field output as measured by a hall device (bottom) **(D)**.

Transcranial magnetic stimulation (TMS) has excellent potential for modulating human brain plasticity; however, the cellular and molecular mechanisms underlying TMS-induced plasticity remain poorly understood. Rodent models of TMS play a significant role in understanding TMS-induced plasticity mechanisms as they offer a more direct measure of TMS-induced synaptic and non-synaptic plasticity (Tang A. et al., 2015). However, one of the main limitations to rodent models of TMS is the lack of rodent-specific TMS stimulator coils. For example, most rodent studies use commercial human coils that are larger than the rodent brain, such as “small” figure of eight (Vahabzadeh-Hagh et al., 2011; Hoppenrath and Funke, 2013) or round coils (Gersner et al., 2011). While the use of such coils allows for stimulation at high intensities used in humans (1–2 T), they lack the equivalent spatial resolution (Weissman et al., 1992) (**Figure 1A**). Offsetting coil position can achieve greater stimulation focality (Rotenberg et al., 2010; Vahabzadeh-Hagh et al., 2011); however, an alternative approach for rodent TMS is to scale-down coil size to improve focality. Whilst recent work has shown that coil size can be dramatically reduced and maintain high intensity capabilities, it still results in relatively unfocal stimulation (Parthoens et al., 2016). In contrast, compromising stimulation intensity for greater focality, rodent-specific coils (circular, 8 mm outer diameter, ~12 mT; **Figure 1B**) have recently been shown to induce structural and molecular plasticity in midbrain and cortical brain regions of mice (Rodger et al., 2012; Makowiecki et al., 2014). However, the effects induced by low intensity TMS may not be representative of the changes produced by high intensity stimulation used in human TMS studies (Grehl et al., 2015).

Thus, there is further need to develop a small animal coil that can deliver TMS at higher intensities, whilst maintaining a good degree of spatial resolution (i.e., focality). However, maintaining high stimulation intensities in small coils has physical constraints such as increased thermal and mechanical stress (Cohen and Cuffin, 1991). The stimulation intensities that can be reliably delivered in an experimental setting by rodent-specific coils have yet to be explored. Here we describe two novel rodent-specific TMS coils that deliver stimulation at modest intensities (~100 mT) to the rodent brain whilst maintaining the spatial resolution of previous low-intensity rodent coils. These small coils provide an alternative approach to the use of non-focal high intensity human coils or focal low-intensity rodent coils, to investigate TMS neuromodulation in rodents.

## Materials and Methods

### Coil and Stimulation Parameters

Two custom circular coils of the same dimensions (8 mm height × 8 mm outer diameter) with either an air or iron core were constructed (**Figure 1C**). Insulated copper wire (0.125 mm diameter, Brocott UK, Yorkshire, UK) was wound (780 turns) around a steel or plastic bobbin (inner diameter 4 mm and outer diameter 8 mm). Coils were wound with a fine wire coil-winding machine (Shining Sun SW-202B, Taipei, China).

Stimulation parameters were controlled by a waveform generator (Agilent Technologies 335141B, CA, USA) connected to a bipolar voltage programmable power supply (KEPCO BOP 100-4M, TMG test equipment, Melbourne, Australia). Current in the coil flowed in a direction that induces an anterior to posterior current across the left rat motor cortex (i.e., posterior to anterior in the coil). Experiments were conducted at 100% of the maximum power supply output (100 V) using custom biphasic waveforms (400 μs rise, 400 μs fall, and 100 μs rise, **Figure 1D**) (Agilent Benchlink Waveform Builder, CA, USA).

### Magnetic Field Decay and Measurements

We used a Hall Effect probe to measure the magnetic field magnitude generated by the coils. Coils were fixed to a stereotaxic frame and manipulated around the Hall Effect probe (Honeywell SS94A2D, NJ, USA). Measurements of single pulse stimulation were taken in the perpendicular (*xy*) and parallel (*z*) axes relative to the main axis of the coil. Due to the axial symmetry of the circular coil, measurements in the *x* axis also represent the *y* axis and are therefore referred to as *xy*. Coil centers were positioned directly above the Hall Effect probe (*xy*, *z* = 0 mm) and repositioned independently at 1 mm increments to a maximum distance of 10 mm in each axis (*xy*_max_ = +10 mm and *z*_max_ = +10 mm). The peak Hall Effect voltage from the rising phase of the biphasic pulse was recorded for 4 pulses at each coordinate and averaged to obtain mean field strength as a function of position. Hall Effect voltages were recorded and analyzed with data acquisition software (Labchart 6, ADI instruments, Sydney, NSW, Australia).

Here we define magnetic field focality as the distance at which the magnetic field is reduced to 50% of the peak.

### Field Strength during 1 and 10 Hz Stimulation

Magnetic field measurements (with the Hall Effect probe) were averaged across the first and last 10 pulses of a 600-pulse train delivered at 1 and 10 Hz, and stability defined as the ratio of these averages expressed as a percentage. Stability % = (Mean magnetic field of the last 10 pulses/Mean magnetic field of the first 10 pulses) × 100.

### Temperature Measurements

Coils were fixed to a K type thermocouple (-40 to 260°C, Dick Smith Electronics Q1437, Perth, WA, Australia) and temperature recordings taken every 50 pulses during the 1 and 10 Hz protocols.

### Sound Measurements

Attempts were made to measure the sound intensity/sound pressure level (SPL) of the brief clicks emitted by the coils undergoing 1 and 10 Hz rTMS using a 1/2” condensor microphone (Bruel and Kjaer Type 4134, Sydney, Australia) placed as close as possible to the coil. The microphone was calibrated using a Bruel and Kjaer Type 4231 calibrator. The output of the 1/2” microphone was viewed directly on an oscilloscope screen (Rigol DS1052E 50 MHz, Measurement Innovation, Perth, WA, Australia). It was found that there was a major artifact in the microphone output that was induced by the magnetic field from the coil and this could not be eliminated by shielding. This induced artifact was critically dependent on the spatial relationship between the TMS coil and the recording microphone, with the smallest artifact being present when these were at right angles to each other. Under these circumstances, the signal from the microphone (presumably a mixture of induced artifact and real acoustic signal) had a peak amplitude that corresponded to approximately 75 dB SPL (re 20 μPa). Because there was no way of separating artifact and acoustic signal in this method, this was thought to be an overestimate of the real sound pressure of the acoustic clicks emitted by the coil. A bioassay method was then used, using two normal hearing human listeners. The sound from the coil inserted into the external ear canal of one ear was matched in apparent loudness to a brief click presented to the other ear using calibrated custom sound generating equipment described in detail elsewhere (Mulders et al., 2011). The duration and spectral content of the clicks were adjusted to match as closely as possible the clicks emitted by the TMS coil.

### Finite Element Modeling

Finite element modelling (FEM) was performed on a high throughput computer cluster consisting of 423 nodes and 4,296 processors using the commercially available Multiphysics 5.0 AC/DC module (COMSOL, Burlington, NJ, USA) to give a general estimate of the induced electric field strength within the animal’s brain tissue during the magnetic stimulation. The geometry of the model was based on the coil used empirically, specifically a multi-turn circular copper wire coil (780 turns, inner diameter of 4 mm, wire diameter of 0.125 mm) containing a soft iron (with losses) core. Only the iron core coil was modeled, as this produced the greatest magnetic field and had better magnetic field stability during rTMS (see RESULTS below). Simulations were performed by driving the coil with a 100 V input (d*I*/d*T* = 1.83 mA/μs) in the frequency domain, using the rise time frequency of the biphasic pulse (2.5 kHz). This method is similar to that used in studies modeling magnetic stimulation of neural tissue, their methods section reviews the equations used for magnetic and electric fields in COMSOL (Bonmassar et al., 2012; Gasca, 2013). Modeling of the electric field was performed using a simplified geometry (Gasca, 2013) of the rat brain, an ellipsoid of 21 mm × 15.5 mm × 10.75 mm, taken from a rat brain atlas (Paxinos and Watson, 1982). The skull was modeled with a thickness of 0.7 mm, the average depth of the rat skull (Levchakov et al., 2006; O’Reilly et al., 2011). The simulation was performed with the coil positioned 0.25 mm above the center of the skull. Dielectric properties for human gray matter and bone were used, and taken from the Foundation for Research on Information Technologies in Society dielectric tissue properties database (Hasgall et al., 2014). These dielectric properties have been used in previous studies of magnetic stimulation in rodents (Nowak et al., 2011; Gasca, 2013; Crowther et al., 2014), and are shown in **Table 1.** Incorporating the frequency dependence of tissue is an important consideration, as low-frequency properties are controlled by the conduction of electrolytes in extracellular space, while high frequencies initiate several biophysical processes which change the dielectric properties of the tissue (Foster, 2000). Modeling was completed with the magnetic fields (mf) physics interface, and consisted of five domains: the brain, skull, the surrounding air, and a multi-turn coil domain inclusive of the iron core and copper wire. The surrounding air domain was created using a condition that approximates the domain as set to infinity so that boundary conditions do not affect the solution. Geometry was discretized to the “extra fine” mesh setting with a swept mesh for the infinite air domain and a boundary condition mesh set around the iron core.

**Table 1:** Dielectric properties used in modeling

	Relative permititvity	Relative permeabilty	Conductivity
Air	1	1	1
Soft Iron (with losses)	1	4.0 × 10^3^	1.12 × 10^7^
Copper	1	1	5.998 × 10^7^
Brain tissue	6.10 × 10^4^	1	1.06 × 10^–1^
Skull	1.53 × 10^3^	1	2.03 × 10^–2^

To compare our iron-core rodent coil with a commercial coil, we ran an additional FEM model on the Magventure MC-B65 butterfly coil placed 7 mm above the ellipsoid rat brain model (as described above). The butterfly coil was modeled similarly to other papers but with parameters specific to the Magventure coil, as two sets of five concentric wires with diameters from 35 to 75 mm, spaced 5 mm apart, and placed 7 mm from the skull (due to the plastic casing; Thielscher and Kammer, 2004; Salvador et al., 2015). The coil input was set at 70% of the maximum stimulator output (MSO ; d*I*/d*T* of 112 A/μs).

### Anesthesia and Electromyography

To determine whether the modest intensities of the rodent-specific coils are suitable for neuromodulation, we delivered sham stimulation (rodent-coil disconnected from power supply) or 10 Hz rTMS to the primary motor cortex of Sprague–Dawley rats (*n* = 4, 250–400 g males) with the iron-core coil. The iron-core coil was selected as it produced the greatest intensity and reliable stimulation at higher frequencies (see RESULTS below). Animals underwent two motor evoked potentials (MEP) recording sessions (a sham stimulation session and an rTMS session over two consecutive days). Animals were pseudo-randomized into MEP sessions, such that an equal number of animals (*n* = 2) received sham and rTMS in session 1 and 2. Changes in cortical excitability (MEPs) were characterized with single pulse TMS and electromyography (EMG) recordings of the rat forelimb as described by Rotenberg et al (Rotenberg et al., 2010). Briefly, rats were deeply anesthetized (verified by absence of pinch reflex) with an intraperitoneal injection of ketamine–xylazine (50 and 10 mg/Kg respectively, Troy Ilium, Sydney, NSW, Australia) and placed into an electrically grounded stereotaxic frame. The torso and all points of contact (ear bars and nose bar) between the animal and the metal frame were insulated with a thin layer of paraffin film to prevent any electrical conductance between the animal and the stereotaxic frame.

Subdermal needle electrodes (13 mm 27G, Neuro Source Medical, ON, Canada) were inserted into the right brachioradialis muscle (recording electrode) and between the 3rd and 4th digit of the right forepaw (reference electrode). Animals were electrically grounded with a single needle electrode inserted into the base of the tail. EMG signals were amplified (×1000), band pass filtered (0.1–1000 Hz) (World Precision Instruments DAM50 Bio-amplifier, Coherent scientific, Adelaide, SA, Australia) and acquired at a sampling rate of 40 kHz (Powerlab 4/30 ADI Instruments, Sydney, NSW, Australia) with Scope software 4.1.1 (ADI instruments, Sydney, NSW, Australia). EMG recordings were stored for post-hoc analysis (MEP peak–peak amplitudes). Automated calculation of MEP amplitudes were calculated in a 10–20 ms window post single pulse TMS (i.e., MEPs had a latency of 10–20 ms post-stimulus). All procedures were approved by the University of Western Australia animal ethics committee (RA/3/100/1371).

### Single Pulse TMS and rTMS

A MagPro R30 stimulator equipped with a Magventure BC-65 butterfly coil (Magventure, Farum, Denmark) was used to deliver single pulse TMS over the left motor cortex. MEP recordings were rapidly generated at 75% of machine stimulator output immediately before and after sham or rTMS stimulation (an intensity known to produce suprathreshold stimulation in rats anaesthetized with ketamine–xylazine (Vahabzadeh-Hagh et al., 2011). Single pulse parameters consisted of 8 pulses with an inter-stimulus interval of 7 s. Immediately following baseline MEP recordings, the iron-core rodent coil (base wrapped in a thin layer of paraffin to insulate the iron-core from the animal) replaced the figure of 8 coil and was placed on the rat head (lightly touching the skull), such that the coil windings overlaid the left motor cortex. This coil position was chosen as the greatest induced current occurs under the windings and not at the coil center. Stimulation consisted of 3 min of sham or 10 Hz rTMS (total of 1800 pulses). Immediately after stimulation, MEPs were recorded (as described above). We chose to record MEPs immediately following stimulation as studies in humans suggest that effects are maximal within the first 20–30 min following stimulation. In addition, we wished to avoid continuous dosing of anesthetic, which results in fluctuations of cortical excitability that are different for each animal. Therefore to maximize the consistency of MEP measurements between animals, we restricted our MEP measurements to within a 30 min window where cortical excitability and anesthesia depth were stable after a single anesthetic injection (as confimed in sham animals). At the end of each MEP recording session, anesthesia was reversed with an intraperitoneal injection of atipamazole (1 mg/Kg, Troy Ilium, Sydney, NSW, Australia) to increase the survival of the animals. All Experimental procedures were approved by the UWA Animal Ethics Committee (03/100/1371).

### Data Analysis

Statistical analysis was performed with SPSS^®^ (IBM, New York, NY, USA). All means are presented with their respective standard error of the mean.

For magnetic field stability, a multivariate ANOVA was conducted to detect coil type (dependent variable) differences in magnetic field stability at 1 and 10 Hz (independent variables). For MEP amplitudes, a ratio of the mean post stimulation MEP amplitude relative to the mean sham MEP amplitude was calculated and log transformed for analysis. A paired *t*-test was conducted to detect whether rTMS (dependent variable) altered MEP amplitudes (independent variable). We also used 95% confidence intervals to support our use of parametric analysis.

## Results

### Magnetic Field Strength – Peak Values and Decay

**FIGURE 2:**
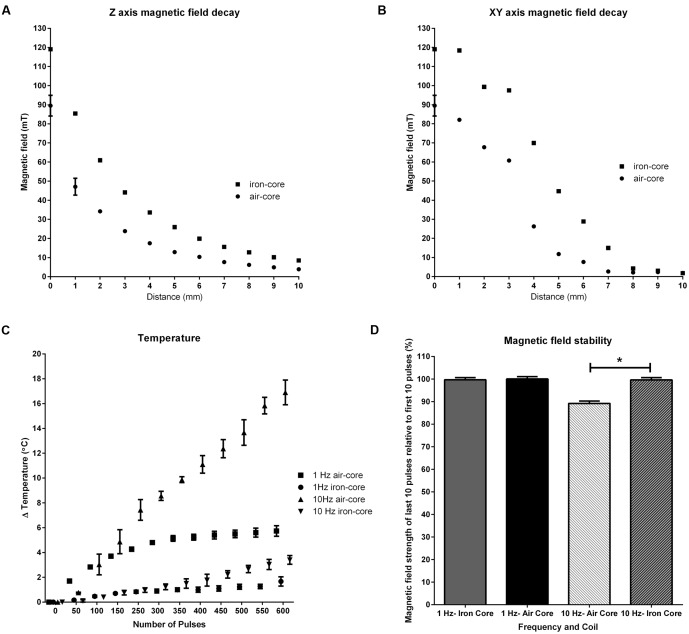
**Characterization of coil properties.** Magnetic field decay in the *z* **(A)** and *xy* **(B)** axes where 0 is the center of the coils, shows the iron-core coil produced a greater peak magnetic field (119.05 mT) than the air-core coil (89.50 mT) with a trade-off of focality. Half-maximum field occurred at ~1.2 mm*_z_*_axis,_ ~3.5 mm*_xy_*_axis_ (air-core) and ~2 mm*_z_*_axis_, ~4 mm*_xy_*_axis_ (iron-core). Changes in the iron-core coil temperature during 600 pulses of 1 and 10 Hz rTMS **(C)** shows tolerable changes in temperature (≤Δ5°C) at both frequencies. 10 Hz stimulation with the air-core coil resulted in a large temperature change (~Δ17.5°C). Magnetic field stability **(D)** shows the iron-core coil shows high stability at both 1 and 10 Hz stimulation. Magnetic field stability for the air-core coil at 10 Hz significantly decreased (^∗^*p* < 0.001) at 10 Hz.

Magnetic field strength in the *xy* and *z* axes is illustrated in **Figure 2.** The iron-core coil produced a greater peak magnetic field (119.05 mT ± 0.42) relative to the air-core coil (89.50 mT ± 6.56) but with decreased focality. Half-maximum field occurred at ~1.2 mm*_z_*_axis,_ ~3.5 mm*_xy_*_axis_ (air-core) and ~2mm*_z_*_axis_, and ~4mm*_xy_*_axis_ (iron-core).

### Changes in Coil Temperature

Temperature measurements over 600 pulses of 1 and 10 Hz stimulation showed frequency and coil type dependent changes (**Figure 2C**). Increases in coil temperature for 1 Hz stimulation peaked at 5.8°C ± 0.40_(Air-Core)_ and 1.67°C ± 0.38_(Iron-Core)_. Peak increases in coil temperature for 10 Hz stimulation were 17.43°C ± 1.07_(Air-Core)_ and 3.57°C ± 0.47_(Iron-Core)_.

Similarly, the time for coil temperatures to return to baseline after 600 pulses of 1 and 10 Hz stimulation showed frequency and coil type dependent changes. Times to return to baseline (min:s) for 1 Hz were 7:42 ± 0:06_(Air-Core)_ and 2:49 ± 0:03_(Iron-Core)_ and 9:39 ± 0.04 and 5:53 ± 0:02_(Iron-Core)_ for 10 Hz.

Change in temperature of the iron-core coil undergoing 1800 pulses of 10 Hz stimulation for neuromodulation and EMG assessment (see below) peaked at 6.8°C ± 0.24.

### Sound Emission from Coils

Measurement of the sound pressure level at the base of the coils undergoing rTMS with a sound level meter sound failed to give an accurate measurement due to the biphasic stimulus artifact induced in the microphone by the rTMS. Using the bio-assay method, an approximation of the peak sound intensity of the TMS clicks emitted by the coils was ~26 dB SPL.

### Magnetic Field Stability

A MANOVA on the magnetic field stability measurements (**Figure 2D**) showed statistically significant coil differences at 10 Hz stimulation (*p* < 0.01) but not at 1 Hz stimulation (*p* = 0.084). At 1 Hz stimulation, both coils showed high stability (100.03% ± 1.03_(Air-Core)_ and 99.70% ± 0.93_(Iron-Core)_) at the end, relative to the beginning, of the stimulation train. However, at 10 Hz stimulation, magnetic field stability was reduced (89.20% ± 1.05 as a result in the reduction in magnetic field intensity towards the end of stimulation) in the air-core coil whereas there was no change in stability for the iron-core coil (99.65% ± 1.02).

### Finite Element Modeling

**FIGURE 3:**
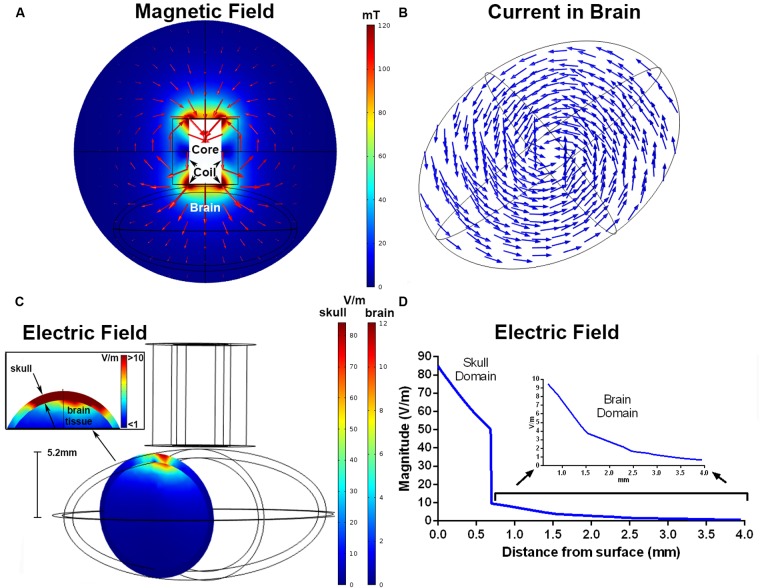
**Finite element modeling of the iron-core coil.** The magnitude of the magnetic field (mT) and magnetic flux density in the *xy* plane **(A)**. The arrows represent the direction of the current density separated in 15 bins. The induced current density within the brain, shown by normalized arrows separated into 12 equal bins for the *xy* grid and 4 in the *z* direction **(B)**. Electric field magnitude (V/m) in a coronal slice of the ellipsoids representing the skull and brain below the coil windings **(C)**. The inset shows an enlarged view of the electric field at the brain and skull interface. The simulated electric field strength within the skull and brain as a function of depth **(D)**. The inset shows electric field strength with the brain domain on a different *y*-axis scale.

Results from the FEM simulation found a magnetic field strength of 115 mT directly below the windings of the coil. The magnetic field distribution (mT) in the *xy* (coronal) plane is shown in **Figure 3A**, and the current density is represented by the arrows in **Figure 3B.** The maximum electric fields simulated within the skull and brains were 85 and 12.7 V/m, respectively (**Figures 3C,D**). These were located below the windings of the coil, similar to the placement of the coil used for the MEP recordings. The estimated electric field was >10 V/m up to a depth of 0.7 mm, >5 V/m to 1.4 mm, and >1 V/m to 3.3 mm.

**FIGURE 4:**
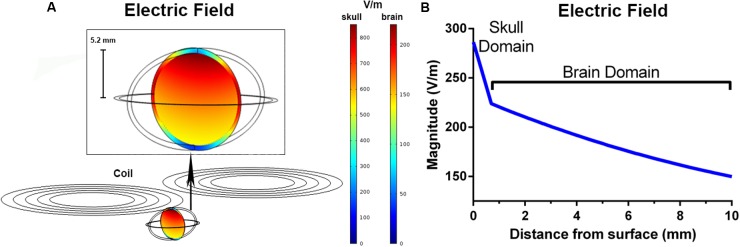
**Finite element modeling of the Magventure BC-65HO butterfly coil.** The induced electric field (V/m) in a coronal slice of the ellipsoid model **(A)**. The simulated electric field strength within the skull and brain as a function of depth **(B)**.

The peak electric fields in the rodent model under the Magventure coil were 1 order of magnitude larger than our rodent coils at 856 V/m in the skull and 224 V/m in the brain (**Figure 4A**). The electric field induced was also larger with an estimated electric field of >150 V/m at a depth of 10mm from the surface (**Figure 4B**).

### 10 Hz rTMS and Cortical Excitability

**FIGURE 5:**
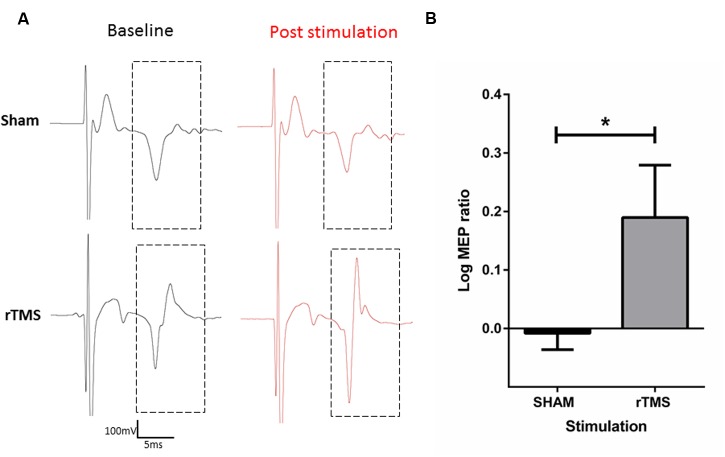
**Characterization of motor evoked potentials (MEP’s) before and after 10 Hz rTMS to the anaesthetized rat motor cortex with the iron-core coil.** Raw electromyography (EMG) traces of sham (top) and active (bottom) rTMS **(A)**. Log_10_ transformation of MEP ratios (post stimulation/baseline) recorded in the right forepaw after 3 min of Sham or 10 Hz rTMS to the left motor cortex. rTMS significantly increased MEP ratios relative to sham stimulation (^∗^*p* < 0.05) **(B)**.

Following sham stimulation, mean MEP amplitude was 98.25% ± 3.207 of the mean baseline MEP amplitude. Following 10 Hz rTMS with the iron-core coil, MEP amplitude was 157.1% ± 15.92 of the mean baseline MEP amplitude. A two-tailed paired *t*-test was conducted on the log_10_ transformed ratios (post stimulation amplitude/baseline amplitude; **Figure 5**) revealed a significant difference between sham and rTMS (mean = 0.198, *SD* = 0.116) conditions; *t* = 3.403, df = 3, *p* = 0.042.

## Discussion

We have developed and characterized two novel rodent-specific TMS coils that can deliver greater stimulation intensities than previous rodent-specific coils similar in size (~12 mT) (Rodger et al., 2012; Makowiecki et al., 2014; Tang A. D. et al., 2015). As expected, the addition of an iron-core increased field strength relative to the air-core coil (Epstein and Davey, 2002) but with a trade-off between greater magnetic field penetration and decreased focality of the iron-core relative to the air-core coil (Deng et al., 2013). Finite element modeling of the iron-core coil undergoing rTMS suggests the induced electric field induced in a simplified rat brain is approximately 1 order of magnitude lower than commercially available human stimulators. Unlike sham stimulation, 10 Hz rTMS with the iron-core coil significantly increased MEP amplitudes relative to baseline.

Our results show that the iron-core coil displays good temperature and magnetic field stability at both 1 and 10 Hz stimulation, whereas, the air-core showed a large increase in temperature and decrease in magnetic field stability at 10 Hz. We attribute the corresponding reduction in field strength to a temperature-related increase in resistance within the copper coil wire. Greater temperature and field stability in the iron-core coils suggest that the core potentially acted as a heat-sink, minimizing heat retention in the copper coil windings. By contrast, temperature increased in the air-core coil most likely because air is a poor conductor of heat. However, it is important that any additional rTMS stimulation protocols be evaluated prior to use, as the efficacy of the iron-core as a heat sink is likely to diminish with higher frequencies (e.g., theta burst protocols), repeated blocks of stimulation or longer durations which may cause excessive heating in the coil with potential harm to the rodents.

Given the greater magnetic field output and thermal stress performance of the iron-core coil, we suggest the iron-core coil is more suitable for use in rodent studies, particularly at high frequency stimulation.

Decreasing coil size has raised the question of stimulation efficiency as smaller coils induce proportionally smaller electric fields. Our calculations are consistent with a model of a commercial TMS stimulator and coil over a mouse brain which found a peak magnetic and electric field of 1.7 T and 132 V/m respectively, approximately 1 order of magnitude larger than our small custom coils (Crowther et al., 2014). Furthermore, our calculations suggest the induced electric field from the iron-core coil results in approximately 10% of the electric field needed for axonal suprathreshold stimulation (100 V/m). Therefore to investigate whether the modest magnetic field/electric field strength delivered by the iron-core coil (~120mT) could induce neuromodulatory effects, we delivered 10 Hz rTMS to a small number of anaesthetized rats combined with EMG recordings to quantify possible changes in MEPs. The iron-core coil was selected as it not only produced the strongest field strength but also showed greater temperature stability and stimulation reliability with high frequency rTMS. Our results showed 10 Hz rTMS significantly increased MEP amplitudes immediately after stimulation, with a mean increase of approximately 57% relative to baseline recordings. These findings are in line with both human (Arai et al., 2007; Jung et al., 2007) and rodent studies (Hsieh et al., 2015) that showed increased MEP amplitudes with subthreshold high frequency rTMS delivered with commercial stimulators and coils. However, although our results provide preliminary evidence that these modest magnetic/electric field intensities can induce neuromodulatory effects in rats, further characterization of changes in cortical excitability and molecular markers are needed. Unlike high intensity rTMS, which involves NMDA and AMPA receptors as elegantly demonstrated by recent publications from the Vlachos and Funke research groups (Labedi et al., 2014; Lenz et al., 2015, 2016), low and moderate intensity rTMS as delivered here is likely to be subthreshold for action potentials, and therefore involve different mechanisms such as changes in intracellular calcium and BDNF levels (Makowiecki et al., 2014; Grehl et al., 2015). By providing a full characterization of the biophysical properties of our small coils, our report will enable future studies to examine in more depth the molecular and cellular mechanisms involved in the induction of cortical plasticity. It will also be important to determine whether the plasticity induced by these small coils is unilateral or bilateral, as well as characterize changes in corticospinal excitability with complete input–output curves, time course of changes and frequency-specific effects.

Approximation of the induced electric field focality of the iron-core coil with FEM modeling showed that the induced electric field peaked below the windings of the coil, and is in line with FEM modeling of commercial coils in spherical head models (Deng et al., 2013). Furthermore, the spread of the electric field was highly localized and undergoes a rapid decay to <1 V/m within millimeters of the peak field. An estimate of stimulation penetration shows that the induced electric field remains above 1 V/m at a distance of 4 mm below the surface of the coil. Accounting for skull thickness (0.7 mm), this equates to an electric field greater than 1 V/m to a depth of ~3.3 mm in the rat brain. This is in contrast to the induced electric field produced with a commercial butterfly coil, which resulted in a greater peak electric field (224 V/m) and more widespread electric field such that the electric field was >150 V/m at a depth of 10 mm from the surface of the brain and encapsulated the entire brain. This is similar to the electric field modeling with the commercial Cool-40 Rat coil, which induces a peak electric field of 220 V/m with a penetration of ≥50 V/m at a depth of ~10 mm (Parthoens et al., 2016). These results suggest that although our coils produce weaker electric fields, they induce more focal stimulation. Given the rapid electric field decay with our coils, it is likely that stimulation is restricted to the cortical and superficial sub-cortical layers of the rat brain (e.g., pyramidal cell layer of the hippocampus) depending on the coil position and orientation. Due to decreased skull thickness and brain size, we expect reduced focality/spatial resolution if used in smaller rodents, such as mice. Whilst this decreases the ability to target specific brain regions, it increases the ability to target deeper brain structures.

A limitation of this study was the need to replace the rodent-specific coil after rTMS with a human figure of 8 coil to induce MEPs. However, due to the subthreshold nature of our rodent-specific coils, eliciting MEPs with a stronger human coil was essential. The use of an unplugged coil to deliver sham is a potential limitation of the study. Whilst the unplugged coil sham maintains the mechanical stimuli of coil placement on the head and background auditory stimuli from the stimulator equipment, it lacks the auditory stimuli of the click sound produced by the coil during active TMS. Approximation of the sound pressure level generated by the air and iron-core coil undergoing 10 Hz rTMS was ~26 dB at the base of the coil. Previous rodent studies suggest that at this intensity, the low frequency sound emitted by the coils is below the hearing threshold of mice (Fernandez et al., 2010) and close to the threshold for rats (Borg, 1982). However, as sound intensity decreases with distance (the inverse square law), it is likely that the ~26 dB at the base of the coil is an over estimation of any sound perceived in the ears of the animal and would be dependent on coil position. Furthermore, it is unlikely that the auditory and small vibration component of active stimulation would induce sensory (e.g., shifts in attention and alertness) and/or placebo (e.g., the belief that one is receiving active stimulation) side effects (Duecker and Sack, 2015), in animals (particularly anaesthetized animals as in this study).

FEM simulations using simplified spherical models are useful when approximating the general electric field properties in neural tissue. However, simplified models come with limitations, which have been addressed in other modeling papers. One of these is that isotropic tissue conductivities are used (Miranda et al., 2013), though a recent paper found no substantial differences in the electric field distribution between models with isotropic versus anisotropic conductivities (Salvador et al., 2015), and another found only weak increases in electric field strength due to the anisotropy of brain tissue (Opitz et al., 2011). Furthermore the electric field estimations (which neglect local maxima at the gyral folds) do not take the radial electric field component into account, and are altered (and likely improved) in more detailed models (Salvador and Miranda, 2009; Thielscher et al., 2011). Whilst the rat and mouse cortex lacks folding and is relatively smooth, estimations of the electric field should be interpreted with care when extrapolating to regions like the cerebellum (where folding does occur in rats and mice) or in the brains of larger rodents such as guinea pigs which have more complex cortices.

## Conclusion

We provide an alternative method to deliver TMS to rodents by constructing small rodent-specific TMS coils capable of delivering modest stimulation intensity whilst maintaining stimulation focality. Our results show different field strengths, penetration, focality, and performance for each coil that need to be considered prior to coil selection. Whilst our coils induce modest magnetic and electric fields, we have shown preliminary evidence that such field strengths can induce neuromodulatory effects. Therefore, we suggest these moderate intensity rTMS coils provide a useful tool for the preclinical investigation of TMS plasticity in rodents.

## Author Contributions

AT and AL conducted the experiments. AT wrote the first version of the manuscript as part of his PhD thesis. AT, AL, AG, RW, RG, AR, JW, and JR designed the study. AT, AL, AG, RW, and JR analyzed the data. All authors revised and proofed the manuscript.

## Conflict of Interest Statement

The authors declare that the research was conducted in the absence of any commercial or financial relationships that could be construed as a potential conflict of interest.
